# Minimal Collagen-Binding Epitope of Glycoprotein VI in Human and Mouse Platelets

**DOI:** 10.3390/biomedicines11020423

**Published:** 2023-02-01

**Authors:** Chao Han, Pengxuan Ren, Medina Mamtimin, Linus Kruk, Edita Sarukhanyan, Chenyu Li, Hans-Joachim Anders, Thomas Dandekar, Irena Krueger, Margitta Elvers, Silvia Goebel, Kristin Adler, Götz Münch, Thomas Gudermann, Attila Braun, Elmina Mammadova-Bach

**Affiliations:** 1 Walther-Straub-Institute for Pharmacology and Toxicology, Ludwig-Maximilian-University, 80336 Munich, Germany; 2 Division of Nephrology, Department of Medicine IV, Hospital of the Ludwig-Maximilian-University, 80336 Munich, Germany; 3 School of Life Science and Technology, Shanghai Institute for Advanced Immunochemical Studies, ShanghaiTech University, Shanghai 201210, China; 4 Department of Bioinformatics, Biocenter, University of Würzburg, 97074 Würzburg, Germany; 5 Department of Vascular and Endovascular Surgery, Heinrich-Heine University Medical Center, 40225 Düsseldorf, Germany; 6 AdvanceCOR GmbH, 82152 Martinsried, Germany; 7 German Center for Lung Research (DZL), 80336 Munich, Germany

**Keywords:** GPVI, collagen, blood platelets, thrombosis, anti-thrombotic therapies

## Abstract

Glycoprotein VI (GPVI) is a platelet-specific receptor for collagen and fibrin, regulating important platelet functions such as platelet adhesion and thrombus growth. Although the blockade of GPVI function is widely recognized as a potent anti-thrombotic approach, there are limited studies focused on site-specific targeting of GPVI. Using computational modeling and bioinformatics, we analyzed collagen- and CRP-binding surfaces of GPVI monomers and dimers, and compared the interacting surfaces with other mammalian GPVI isoforms. We could predict a minimal collagen-binding epitope of GPVI dimer and designed an EA-20 antibody that recognizes a linear epitope of this surface. Using platelets and whole blood samples donated from wild-type and humanized GPVI transgenic mice and also humans, our experimental results show that the EA-20 antibody inhibits platelet adhesion and aggregation in response to collagen and CRP, but not to fibrin. The EA-20 antibody also prevents thrombus formation in whole blood, on the collagen-coated surface, in arterial flow conditions. We also show that EA-20 does not influence GPVI clustering or receptor shedding. Therefore, we propose that blockade of this minimal collagen-binding epitope of GPVI with the EA-20 antibody could represent a new anti-thrombotic approach by inhibiting specific interactions between GPVI and the collagen matrix.

## 1. Introduction

Platelets are small anucleated cells, which play a central role in the maintenance of hemostasis, but in pathological conditions, abnormal platelet functions may trigger thrombosis or bleeding complications [1,2]. During vessel injury, platelets adhere to the damaged endothelium and become activated. This process involves adhesion receptors, including integrins and platelet-specific glycoproteins, that link platelets to extracellular matrix (ECM) proteins, such as collagen, exposed on the surface of the damaged endothelium [1,2,3]. In pathological conditions, the exposure of collagen from the subendothelial matrix of the atherosclerotic vessel wall induces platelet activation and thrombus growth, thereby increasing the risk of thromboembolism, myocardial infarction, and stroke [4,5,6,7]. Collagen fibers are composed of three polypeptide chains, characterized by a repeating Gly-X-Y sequence, where X is frequently proline (P) and Y is 4-R-hydroxyproline (O) [8]. The GPO triplets are essential for ligand binding, activating receptors on the platelet surface. Integrin α2β1, glycoprotein (GP) V, and GPVI can directly bind type I collagen fibers [3,9,10,11]. 

### 1.1. Collagen-Binding Sites of GPVI Receptor

GPVI interaction with collagen regulates platelet adhesion, activation, aggregation, and procoagulant activity that is essential for thrombus growth [9,10]. GPVI belongs to the immunoglobulin superfamily, building a complex with the immunoreceptor tyrosine-based activation motif (ITAM)-subunit FcRγ-chain. GPVI has two extracellular C2-type Ig-like domains (D1 and D2 domains) which are linked to a transmembrane (TM) domain and a short cytoplasmic tail, interacting with Src family kinases, protein kinase C (PKC), and calmodulin [10,11]. The first crystal structure of the D1 and D2 domains of the human GPVI proposed the dimerization site between two β-strands of D2 domains [12]. The amino acid residue in the TM domain of GPVI forms a salt-bridge with the FcRγ-chain, triggering ITAM-mediated signals to the linker adaptor for the T cell (LAT) complex. Interestingly, GPVI dimers bind with high affinity to tandem GPO sequences on the collagen fiber, while GPVI monomers had a lower affinity, indicating that mainly GPVI dimers are involved in collagen-dependent platelet adhesion. Therefore, it was proposed that a functional unit of GPVI is a dimer [13,14,15]. In line with this result, most GPVI receptors on the surface of resting platelets form dimers, and the number of dimers is further increased during platelet activation, promoting collagen-mediated platelet adhesion [13,14]. 

### 1.2. CRP-Binding Sites of GPVI Receptor

Besides collagen, synthetic collagen-related peptides (CRP) can also bind GPVI, mimicking collagen-induced platelet activation [16]. CRP is composed of 10 repeated GPO triplets from collagen. CRP peptides are cross-linked, thereby becoming a potent and selective agonist of the GPVI receptor to induce ITAM-signaling events. Snake venom toxins, such as convulxin and alborhagin, also bind and activate GPVI [17,18,19], but their interaction is different from collagen and does not require GPVI dimerization, suggesting an alternative mode to cluster and activate GPVI on the platelet surface. Convulxin can induce GPVI dimers (α4 and β4 rings) in solution, thereby clustering several copies of GPVI, indicating that these interactions are maintained by multiple binding sites [20]. Amino acid residues of GPVI have been identified as being involved in the recognition and binding of collagen [21,22,23]. However, as described by Smethurst et al, the collagen-binding site may be located at a distant surface from the potential CRP-binding site [21,22]. They proposed that the collagen-binding surface thus appears to be widespread on GPVI, and covers a larger binding surface compared to the CRP-binding site. In other studies, several amino acids have been implicated in both collagen- and CRP-binding, Ref. [23] suggesting an overlapping binding surface, whereas some amino acids are involved in binding of collagen, but not CRP. 

### 1.3. Anti-Thrombotic Approaches Based on the Blockade of GPVI-Collagen Interaction 

GPVI is recognized as a safe anti-thrombotic target, based on the observations that its blockade reduces experimental thrombosis without impairing hemostasis [11]. Although initial phases of arterial thrombosis are regulated by GPVI interactions with collagen, at further steps GPVI stabilizes the growing thrombus through binding to fibrin [24,25,26,27]. Therefore, several GPVI inhibitors and blocking antibodies have been developed and evaluated in preclinical models or clinical trials of patients with thrombotic diseases to target GPVI functions, thereby inhibiting thrombus formation [11,28,29]. However, limited studies are available to map the collagen-binding epitope of GPVI and specifically target only this clinically relevant interaction in platelets. 

In our study, using the crystal structure of the D1 and D2 domains of human GPVI and collagen, we modeled the collagen- and CRP-binding surfaces of the GPVI monomer and dimer, and compared them with other mammalian GPVI isoforms. Using computational modelling and a docking approach, we identified a minimal collagen-binding epitope of GPVI in both human and mouse platelets. Based on these results, we designed a GPVI-blocking antibody (EA-20) which selectively inhibits collagen-mediated platelet adhesion, activation, and aggregation responses, without affecting fibrin–GPVI interaction. 

## 2. Materials and Methods 

### 2.1. Computation Method and EA-20 Antibody Design 

The structure of GPVI, in complex with collagen-peptide (PDB code: 5OU8) [30], was retrieved from the protein structure databank (http://www.rcsb.org; accessed 20 April 2022). The protein structures were prepared by the Protein Preparation Wizard in Maestro (Schrödinger. LLC., Portland, OR, USA) [31] for assigning correct protonation states, formal charges, adding missing residues, and optimizing three dimensional (3D) conformations. Then, ZDOCK server (https://zdock.umassmed.edu/; accessed 20 April 2022) was used [32] to perform docking simulation. The top 10 complex structures were downloaded, and the most possible complex structure was chosen by visual inspection. The predicted complete structure of GPVI (AF-Q9HCN6-F1), by AlphaFold (Hinxton, Cambridge, United Kingdom) [33,34], was aligned to the chosen complex structure. The Protein Preparation Wizard in Maestro minimized the predicted complex structure. The interaction analysis of two proteins was performed by the Protein Interaction Analysis Panel in Maestro. All visualizations were performed using Pymol (Schrödinger. LLC., Portland, OR, USA) [35]. The selected peptide (^48^CQGPPGVDLYRLEKL^62^) from the interacting surface of GPVI is conserved between human and mouse, except that G^53^ is changed to D in mouse. Peptides with wobble amino acids of D/G in the same batch were linked to KLH. Rabbits were immunized to obtain rabbit IgG polyclonal antibody EA-20. The EA-20 antibody was produced and purified using affinity chromatography by Eurogentec, Liege, Belgium.

### 2.2. Homology Modeling and Phylogenetic Analysis

The homology models for the GPVI protein of *Homo sapiens*, *Mus musculus*, *Bos Taurus,* and *Sorex araneus* were obtained using the SWISS MODEL online tool (https://swissmodel.expasy.org; accessed 27 September 2022). The amino acid sequences of GPVI proteins were compared with published sequences using the Basic Local Alignment Search Tool (BLAST) analysis on the website of the UniProt (https://www.uniprot.org/blast; accessed 12 December 2021). The sequences were then aligned by multiple sequence alignment (MSA) package through Clustal-W multiple alignments [36]. The phylogenetic tree was constructed using the maximum likelihood or neighbor-joining tree method with 1000 bootstrap.

### 2.3. Generation of Humanized GPVI Mice

C57BL6/J wild-type (*WT*) and humanized GPVI (*hGP6*) mice were used, and all experiments were performed with 2 to 3 month-old male and female mice. The humanized GPVI mouse line (*hGP6*) was generated by AdvanceCOR GmbH (Martinsried, Germany). Briefly, a chimeric human *GP6* and mouse *Gp6* cDNA was generated, coding the full length of human extracellular domains (D1/D2), which was fused with the mouse GPVI transmembrane domain and short cytoplasmic tail. The ATG-coded exon of the mouse *Gp6* gene was replaced with the chimeric *GP6* cDNA and polyA in embryonic stem cells by homologous recombination, expressing chimeric GPVI proteins by the endogenous mouse *Gp6* promoter. The humanized GPVI mice (*hGP6*) mice were genotyped using the following primers: forward 5′ CGTGGGTGTTAATCCAGCCAATGTA 3′ and reverse 5′ CTATAACTCCAGCTCCAAAGAATCCAATT 3′, and gene specific products were amplified by PCR (wild-type: 435 bp; *hGP6*: 517 bp) (Supplementary Figure S1). 

### 2.4. Platelet Isolation 

Mice were anesthetized and 700 µL of whole blood was collected in a reaction tube containing 300 μL diluted heparin with Tyrode’s buffer (20 U/mL, pH 7.3, Sigma, Taufkirchen, Germany). Human blood was collected in 10 mL monovet tubes containing 3.2% citrate (Sarstedt, Nümbrecht, Germany) from veins of healthy volunteers who had not taken any antiplatelet medication in the preceding 2 weeks. Mouse and human platelets, and platelet-rich plasma, were isolated from whole blood as previously described [37,38].

### 2.5. Platelet Aggregation

Light transmission of 40 µL platelet suspension, supplemented with 160 µL Tyrode’s buffer (final concentration: 200 × 10^3^ platelets/μL) containing 100 μg/mL fibrinogen and 2 mM Ca^2+^, and platelet aggregation was monitored over 10 min using a four-channel APACT4004 aggregometer (Labitec, Ahrensburg, Germany). Aggregation studies with ADP were performed in platelet-rich plasma (PRP). Washed platelets and PRP were incubated with 20 µg/mL EA-20, control IgG antibody and/or seroblock (Bio-Rad, Feldkirchen, Germany) and/or Losartan (50 µM, Sigma, Taufkirchen, Germany) for 5 min. Platelet aggregation was induced by the addition of type I collagen (Col-I, Haemochrom Diagnostica GmbH, Essen, Germany), collagen-related peptide (CRP, Cambcol, Cambridgeshire, UK), thrombin (Roche, Grenzach-Wyhlen, Germany) and thrombin receptor activator for peptide 6 (TRAP-6, Haemochrom Diagnostica GmbH, Essen, Germany), and adenosine diphosphate (ADP, Sigma, Taufkirchen, Germany).

### 2.6. Static Adhesion Assay

Glass coverslips were coated with 50 µg/mL Col-I (Haemochrom Diagnostica GmbH, Essen, Germany) or the mixture of 200 µg/mL human fibrinogen (Sigma, Taufkirchen, Germany) and 0.1 U/mL Thrombin (Sigma, Taufkirchen, Germany), kept overnight at 4 °C, and blocked with PBS 1x 0.5% BSA for 30 min. Washed mouse and human platelets (10^7^ platelets/coverslip) were suspended in Tyrode’s buffer and incubated with 20 µg/mL EA-20 or control IgG antibody for 5 min, then allowed to adhere to the coated surfaces at 37 °C. Non-adherent platelets were washed away, and adherent cells were fixed with 4% PFA in PBS 1x, and then stained with an anti-CD41-Alexa-488 antibody (Biozol, Eching, Germany). Coverslips were mounted and images were obtained from ten different collagen- or fibrin-containing microscopic fields for each sample, using immunofluorescence confocal Zeiss microscope (Carl Zeiss, Oberkochen, Germany). Platelet adhesion was calculated using ImageJ software (Bethesda, MD, USA).

### 2.7. Platelet Adhesion under Flow

Mouse or human blood samples were collected as indicated above (Section 2.4) and used for ex vivo flow adhesion assay in a flow chamber system (Maastricht Instruments, Maastricht, The Netherlands). Rectangular cover slips (24 × 60 mm) were coated with 200 μg/mL fibrillar type I collagen (Haemochrom Diagnostica GmbH, Essen, Germany) and blocked with 1% BSA in PBS 1x for 1 h. Anticoagulated mouse or human whole blood was incubated with 2 µg/mL anti-CD42b-DyLight-488 antibody (Emfret Analytics, Eibelstadt, Germany) or 10 µM mepacrine salts (Sigma, Taufkirchen, Germany) and 20 µg/mL EA-20 and/or control IgG antibody for 5 min, then perfused over collagen-coated coverslips through a transparent flow chamber at a shear rate of 1000 s^−1^. Phase-contrast and confocal fluorescence images were obtained from 10 different areas, using an Axiovision epifluorescent and confocal Zeiss microscope (Carl Zeiss, Oberkochen, Germany), to analyze surface coverage using ImageJ software (Bethesda, MD, USA). 

### 2.8. Measurement of Soluble GPVI

For GPVI measurement, 96-well microtiter plate wells (Sarstedt, Nümbrecht, Germany) were coated with 10 µg/mL capturing rat anti-GPVI antibody 1A5 (AdvanceCOR GmbH, Martinsried, Germany) in a coating buffer (0.05 M bicarbonate, pH 9.6), kept overnight at 4 °C. Wells were washed three times with washing buffer (0.01 M sodium phosphate, 0.15 M sodium chloride, pH 7.4, PBS 1x 0.1% Tween-20 (PBS-T) and blocked with 3% milk PBS-T (blocking solution) for 1 h at room temperature, then washed three times with PBS-T before the addition of plasma samples in triplicates. The measurement included standards consisting of recombinant His-tagged GPVI ectodomain (0–100 ng/mL, final concentration) in 1% BSA (Sigma, Taufkirchen, Germany). Plasma samples were diluted in PBS-T 1% BSA (Sigma, Taufkirchen, Germany) to be directly comparable to standards. After 1 h incubation at room temperature, wells were washed three times with PBS-T, and 100 ng/mL rat anti-GPVI antibody 4C9-DIG (AdvanceCOR GmbH, Martinsried, Germany) was added in blocking solution and incubated again for 1 h. After three consecutive washes, anti-Digoxigenin-POD (poly) Fab fragment (Roche, Grenzach-Wyhlen, Germany) was added into the wells and incubated for 1 h. After three washing steps, the wells were incubated with TMB substrate (Life Technology, Darmstadt, Germany) for 15 min, and the reaction was stopped by adding 1 M H_2_SO_4_. The absorbance was measured at wavelength 450 nm using a Tecan microplate reader (Tecan, Männedorf, Switzerland).

### 2.9. Statistical Analysis 

The statistical significance of results was analyzed using the GraphPad Prism program, version 8.0 (Prism, GraphPad). The Shapiro–Wilk normality test was used to analyze the normality of the data. The statistical difference of the mean was analyzed using the Student unpaired two-tailed *t* test or 1-way ANOVA and Tukey’s post hoc test. For data not following a Gaussian distribution, the non-parametric Mann–Whitney test was used. *p*-values < 0.05 were considered as statistically significant (* *p* < 0.05; ** *p* < 0.01; *** *p* < 0.001; **** *p* < 0.0001) and *p*-values > 0.05 as not significant (ns).

## 3. Results

### 3.1. Collagen and CRP-Binding with GPVI Monomer in Mammalian Species

**Figure 1:**
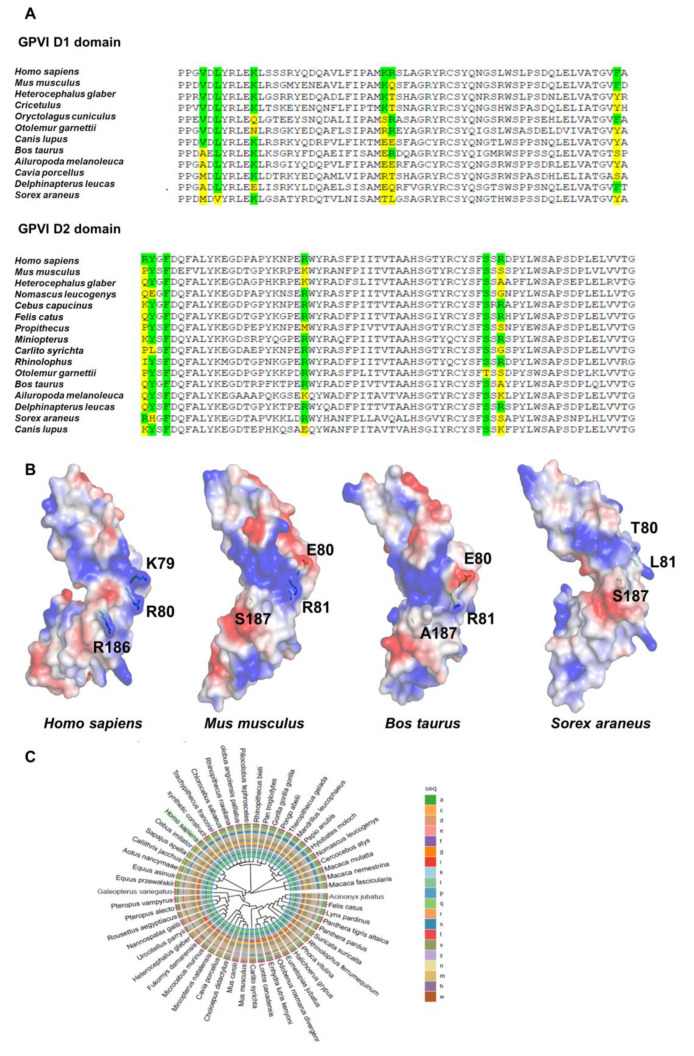
Conserved regions of GPVI in mammalian species. (**A**) The multiple sequence alignment of mammalian GPVI. D1/D2 domain of human GPVI was compared to other mammalians. The conserved amino acid residues involved in collagen- and CRP-binding are indicated in green. The amino acid residues involved in collagen- and CRP-binding which are not fully conserved between mammalian species are indicated in yellow. (**B**) 3D homology models for *Homo sapiens*, *Mus musculus*, *Bos Taurus,* and *Sorex araneus*. The conserved amino acid residues of human (*Homo sapiens* K79, R80, and R186) and mouse GPVI (*Mus musculus* E80, R81, and S187) are shown as sticks; those of bovine (*Bos Taurus* E80, R81, and A187) and sorex GPVI (*Sorex araneus* T80, L81, and S187) are highlighted in cyan and orange, respectively. Electrostatic surface potentials are shown in red and blue for negative and positive charges, respectively, and white for neutral residues. (**C**) The neighbor joining tree of the GPVI D1 domain indicating the phylogenetic relationship between mammalian GPVI isoforms.

Site-directed mutagenesis studies have identified several important amino acids located in the D1/D2 domain of human GPVI, which are involved in collagen- or CRP-binding (green box, Figure 1A). Using the online computational tool BLAST for multiple sequence alignment, we further analyzed the D1/D2 domain of human GPVI and compared it to that of other mammalians. Interestingly, we found that many investigated key amino acid residues, important for collagen- or CRP-binding, are not fully conserved between mammalian species (yellow box, Figure 1A). Definitively, an ancient mammalian species *Sorex araneus* showed the most variable amino acid residues within the D1/D2 domain. However, important amino acid sequences of the D1/D2 domain, such as cysteine residues, which determine the secondary structure of the protein, showed high conservative sequences between species (Figure 1A). Next, we used 3D homology models for *Homo sapiens*, *Mus musculus*, *Bos Taurus,* and *Sorex araneus,* constructed using a SWISS-MODEL online tool (https://swissmodel.expasy.org; accessed 27 September 2022). Similar to the multiple alignment data, the following amino acid residues were indicated as probably covering the collagen or CRP-binding surface: K79, R80, and R186 for *Homo sapiens* (human GPVI); E80, R81, and S187 for *Mus musculus* (mouse GPVI); E80, R81, and A187 for *Bos Taurus* (bovine GPVI); and T80, L81, and S187 for *Sorex araneus* (sorex GPVI) (Figure 1B). Analysis of the 3D structure of the GPVI monomer showed that the main structure of GPVI is similar between species, but amino acid changes are observed on the collagen- and CRP-binding surface, which clearly changes the electrostatic surface potentials (red color, negative; blue color, positive; white color, neutral residues) (Figure 1B). This result suggests that the biochemical properties of GPVI that allow binding of collagen or CRP may be different between mammalian species. Next, the phylogenetic relationship between mammalian GPVI isoforms was functionally characterized and placed in a single clade related to order. Over all, this result showed an evolutionally conserved relation between GPVI across numerous mammalian species (Figure 1C; Supplementary Figures S2 and S3).

### 3.2. Collagen-Binding with GPVI Dimer in Humans 

**Figure 2:**
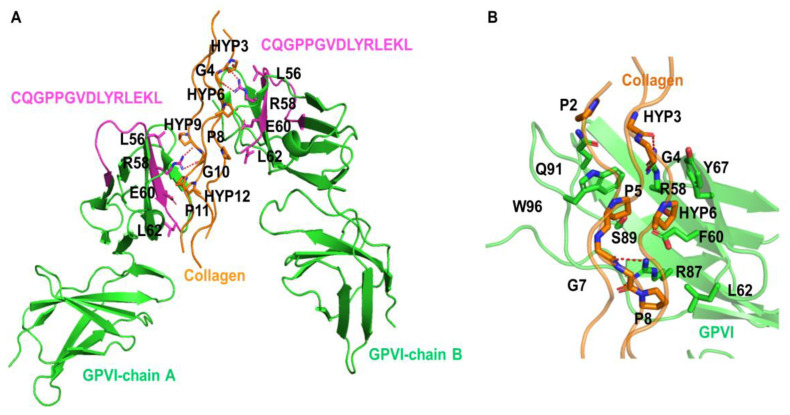
Binding mode of collagen with GPVI. (**A**) The docking simulation of collagen and GPVI dimer. The interaction between collagen and the blocking peptide residues in GPVI. The complex structure (PDB code: 5OU8) of GPVI and collagen-peptide was retrieved from the protein structure databank. GPVI is shown in green; peptide residues in GPVI and collagen are shown in magenta and orange, respectively. (**B**) The zoomed-in interaction between collagen and peptide residues in GPVI. The interaction residues are depicted as sticks. Red dash lines indicate hydrogen bonds between residues.

Experimental evidence suggests that only GPVI dimers strongly bind collagen, whereas monomeric GPVI only weakly binds this ligand [15]. Using a competitive platelet aggregation assay, it has been shown that soluble dimeric GPVI-Fc fusion protein inhibits collagen-mediated platelet aggregation, while soluble monomeric GPVI-Fc fusion protein has only a minor, or no effect, on collagen-binding [15]. To prove this concept, we performed a molecular docking study using the recently published crystal structure of the human GPVI dimer and human collagen to validate the binding surface of GPVI (Figure 2A). Collagen structures were docked between the dimer of GPVI, which is consistent with the experimental structure (PDB code: 5OU8). Several amino acid residues were depicted in the D1 domain of the human GPVI dimer (green line: R58, E60, L62, Y67, R87, S89, Q91, W96) which possibly interact with collagen residues (orange line: Hyp3, Hyp6, P5), thereby stabilizing the protein complex (Figure 2A,B). The predicted collagen-binding site of the human GPVI dimer overlapped with the mouse GPVI dimer. Of note, the amino acid residues of GPVI, which are within the distance of 3 Å with those of collagen, are considered as interacting residues. The list of interacting amino acid residues, as well as the corresponding distances, is provided in Table 1.

### 3.3. EA-20 Antibody Inhibits GPVI-Mediated Platelet Activation and Thrombus Formation on Collagen, but Not on Fibrin

**Figure 3:**
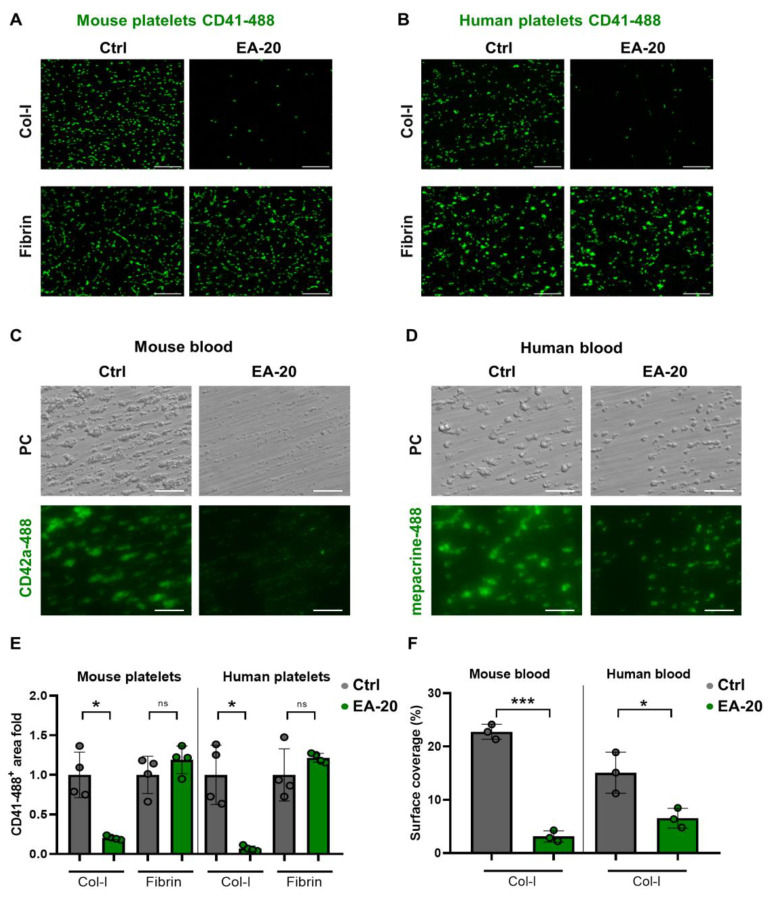
EA-20 antibody selectively impairs platelet adhesion and thrombus formation to Col-I-coated surfaces under static and flow conditions. Representative immunofluorescence and phase contrast microscopy images of mouse and human platelets adhering to Col-I and fibrin under (**A**,**B**) static and (**C**,**D**) flow conditions, respectively. Scale bar: 20 μm. Washed (**A**) mouse and (**B**) human platelets were allowed to adhere to Col-I and fibrin-coated glass coverslips in the presence of an IgG control (Ctrl) and or EA-20 antibody. After 1 h, adherent cells were fixed, stained with an anti-CD41-488 antibody, and observed by immunofluorescence confocal microscopy. Anticoagulated mouse (heparinized, **C**) and human (citrated, **D**) whole blood, stained with anti-CD42a-(DyeLight)-488 or mepacrine salts, were perfused over Col-I-coated glass slides at 1000 s^−1^ shear rate in the flow chamber in the presence of control IgG (Ctrl) and or EA-20 antibody and monitored for 5 min. (**C**,**D**) Representative phase contrast (upper panel) and fluorescence microscopy (lower panel). PC (Phase contrast). Scale bar: 10 μm. (**E**,**F**) Quantification of surface coverage of fluorescent signal corresponding to the amount of adherent platelets and or thrombus formation. (**E**,**F**) Mean ± standard deviation (SD), Ctrl (IgG control antibody) and EA-20. (**E**,**F**) Each point represents one individual mouse or healthy human donor. (**E**,**F**) Mann–Whitney U test, * *p* < 0.05; unpaired *t* test, *** *p* < 0.001 and * *p* < 0.05; ns, not significant.

To confirm our bioinformatic results experimentally, we designed a rabbit polyclonal peptide antibody (clone name: EA-20) which recognized a linear epitope on the surface of GPVI (^48^CQGPPGVDLYRLEKL^62^) and potentially inhibits the interaction between GPVI and collagen (Figure 2A,B, Table 1). Since the peptide sequence is conserved between human and mouse GPVI (only ^53^G changed to D in mouse), we synthetized peptides with wobble amino acids of D/G in the same batch, linking KLH and immunized rabbits to obtain a rabbit IgG polyclonal antibody (EA-20) which was later affinity purified. We studied the effects of the EA-20 antibody on both mouse and human platelets responding to collagen and fibrin, using static and flow adhesion assays. First, washed mouse and human platelets were allowed to adhere to the collagen and fibrin-coated surfaces under static conditions (Figure 3A,B). We found that the EA-20 antibody significantly reduced the adhesion of mouse and human platelets to collagen (Figure 3A,B,E). In sharp contrast, no differences were observed on the fibrin-coated surface (Figure 3A,B,E), indicating that the EA-20 antibody can selectively block collagen interaction with GPVI in both species. Next, we analyzed the blocking effect of the EA-20 antibody under thrombus formation on the collagen-coated surface, at shear rate of 1000 s^−1^, using human or mouse whole blood in the flow chamber. Mouse blood treated with EA-20 antibody exhibited strongly reduced platelet adhesion and impaired thrombus formation (Figure 3C,F). Similar results were obtained using human blood, although the inhibition of thrombus formation was less severe than that observed with mouse platelets (Figure 3D,F). It is important to note that thrombus formation was not completely abolished, indicating a partially defective thrombus formation, possibly maintained by GPVI and fibrin interaction. 

**Figure 4:**
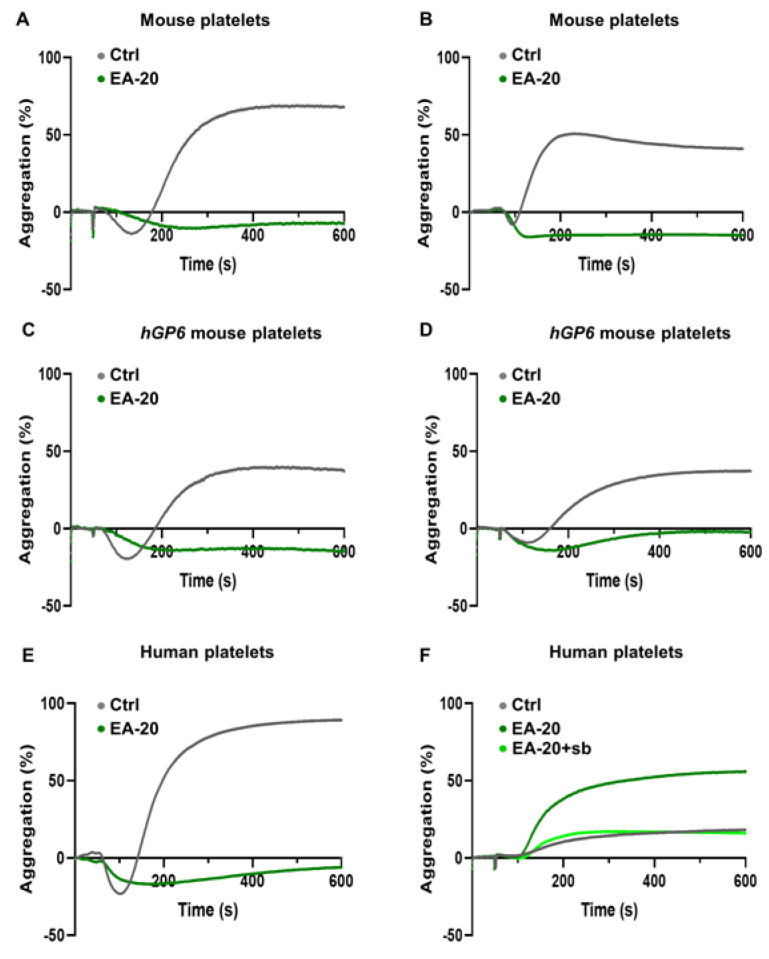
EA-20 antibody impairs aggregation of mouse and human platelets in response to Col-I and CRP. Washed mouse and human platelets were stimulated with Col-I (**A**,**C**,**E** Col-I 2.5 µg/mL) and CRP (**B**,**D** CRP 0.2 µg/mL and **F** CRP 0.4 µg/mL) in the presence of control IgG (Ctrl) or EA-20 antibody. Experiments were performed using (**A**,**B**) *WT* mouse, (**C**,**D**) humanized GPVI (*hGP6*) mouse, and (**E**,**F**) human platelets; sb: seroblock. Platelet aggregation was measured using light transmission aggregometry and recorded for 10 min. Representative aggregation curves are shown. Statistical analysis is shown in Supplementary Figure S4.

Using mouse and human washed platelets, aggregation responses to collagen were also severely impaired (Figure 4A,E; Supplementary Figure S4A,E). In the humanized mouse model of GPVI, expressing only the human GPVI isoform on the mouse platelet surface, the EA-20 antibody was tested and aggregation response to collagen was also reduced (Figure 4C; Supplementary Figure S4C). Interestingly, CRP-induced aggregation response was strongly impaired in the presence of the EA-20 antibody in humanized GPVI or wild-type mouse platelets (Figure 4B,D; Supplementary Figure S4B,D), but, in sharp contrast, abnormal hyper-aggregation was observed in human platelets when a low dose of CRP was applied (Figure 4F; Supplementary Figure S4F). However, such an aggregation effect was not observed in response to a low dose of collagen (Supplementary Figure S5). Human immunoglobulin G (IgG) Fc receptor, gamma IIa (Fc**γ**RIIa) receptor, is expressed in human platelets and fully activated by IgG. However, the Fc**γ**RIIa receptor is not expressed in mouse platelets. Therefore, we assumed that the abnormal response to CRP was triggered by rabbit IgG of the EA-20 antibody, activating Fc**γ**RIIa receptor signaling, a mechanism that is absent in mouse platelets. To prove this concept experimentally, human seroblock was used to block IgG-mediated clustering and activation of the Fc**γ**RIIa receptor. After CRP activation, treatment with seroblock could revert EA-20-induced hyper-aggregation of human platelets (Figure 4F; Supplementary Figure S4F). Of note, the EA-20 antibody did not influence the platelet aggregation response to non-GPVI agonists, such as thrombin, TRAP-6, and ADP (Supplementary Figures S6 and S7). Altogether, these results indicate that the EA-20 antibody selectively inhibits the interaction between GPVI and collagen, but does not influence other platelet receptor functions or fibrin binding to GPVI.

### 3.4. Effect of EA-20 Antibody on GPVI-Clustering and Shedding in Human Platelets 

**Figure 5:**
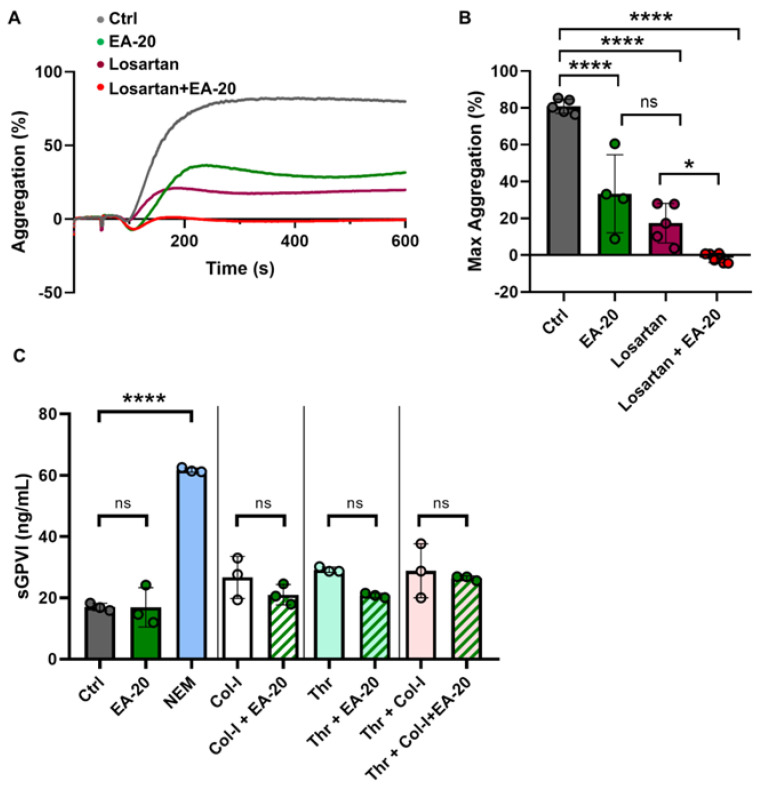
EA-20 antibody does not induce clustering or shedding of GPVI. (**A**,**B**) Washed human platelets were stimulated with Col-I (2.5 µg/mL) in the presence of control IgG (Ctrl) or EA-20 antibody and/or Losartan (50 µM). Platelet aggregation was measured using light transmission aggregometry and recorded for 10 min. Representative aggregation curves are shown. (**C**) Human PRP were stimulated with Col-I (10 µg/mL), thrombin (1U), a combination of Col-I (10 µg/mL) and thrombin (1U), or GPVI shedding-inducing-reagent NEM (2 mM), in the absence or presence of EA-20 antibody. After 1 h incubation, platelet-free plasma (PFP) was collected and soluble GPVI (sGPVI) levels, indicative of platelet GPVI-shedding, were determined using ELISA assay. Mean ± standard deviation (SD), Ctrl (IgG control antibody), and EA-20. Each point represents one individual (**B**,**C**) healthy human donor. 1-way ANOVA and Tukey’s post hoc test, * *p* < 0.05, **** *p* < 0.0001; ns, not significant.

Previously, Losartan has been used to inhibit collagen-induced GPVI activation by reducing the clustering of GPVI on the platelet surface [39]. To study whether EA-20 may inhibit GPVI clustering as well, human platelets were activated by collagen in the presence or absence of Losartan and the EA-20 antibody (Figure 5A,B). We found a strong inhibition of aggregation response to collagen when human platelets were treated with both Losartan and EA-20 (Figure 5A,B), indicating that EA-20 antibody does not inhibit GPVI clustering, and only affects collagen binding. To investigate whether the EA-20 antibody may induce GPVI shedding from the surface of human platelets, platelet-rich plasma samples were collected from healthy donors and stimulated with collagen (Col-I), thrombin (Thr), and the thiol-modifying agent N-ethylmaleimide (NEM) as a positive control (Figure 5C). All these treatments have previously been shown to induce GPVI shedding from the human platelet surface, thereby increasing soluble GPVI levels (sGPVI) in the plasma [40]. Using a standardized GPVI ELISA, we found that collagen, thrombin, and NEM treatment increased the level of sGPVI, while co-incubation of platelets with the EA-20 antibody did not further increase the amount of sGPVI in the plasma (Figure 5C), indicating that EA-20 antibody-mediated inhibition of platelet aggregation is not mediated by GPVI shedding.

## 4. Discussion

The biochemical properties of GPVI that allow binding of CRP were investigated in human and mouse platelets, and results showed that the CRP-binding affinity of human GPVI was significantly higher than mouse GPVI [21]. Similar to other studies, we could confirm that the human and mouse CRP-binding surface of the D1 domain is variable; several amino acids were changed during evolution, thereby changing the electrostatic surface potential, which possibly modifies the ligand-binding affinity of GPVI. In ancient mammalian species, such as *Sorex araneus,* we detected the most variable peptide sequence of the D1 domain (^48^CQGPP**DM**D**V**YRLEKL**G**S**AT**Y**R**DQ**T**VL**N**I**S**AM**TLGS**^82^), which is definitively involved in collagen and CRP-binding of GPVI in many species, including human and mouse. Therefore, we speculate that this modification in *Sorex araneus* may strongly reduce the collagen-binding affinity of GPVI. However, it may not influence the interaction with other GPVI ligands, such as laminin or fibrin, because these amino acid changes do not influence the main structure of GPVI, as we observed in 3D GPVI models of human, bovine, mouse, and sorex species. 

JAQ-1 is an anti-mouse GPVI monoclonal antibody, which inhibits the major collagen and CRP-binding site in platelets [41]. Interestingly, JAQ-1 showed specific interaction with mouse and human GPVI, but, unexpectedly, it cannot bind platelets isolated from different mammalian species, such as rat, rabbit, guinea pig, dog, and swine, experimentally supporting our finding that the collagen and CRP-binding surface of GPVI diverged during evolution [42]. Although the JAQ-1 antibody recognized an epitope on hGPVI, it also induced an abnormal aggregation response in human platelets on collagen [42]. 

Unhydroxylated collagen, isolated from transgenic plants, or unhydroxylated CRP (GPP10), is not able to bind or activate platelets [43]. A single amino acid substitution of GPVI at position K^61^ (^48^CQGPPGVDLYRLE**K**L^62^) could increase the binding affinity of GPVI to hydroxylproline-containing motifs, indicating that hydroxylation of collagen fibers is essential for GPVI interaction. Interestingly, in many mammalian species, this amino acid was spontaneously mutated from K to Q, or E or N, during evolution, supporting the idea that this amino acid substitution may increase the collagen-binding affinity of GPVI in these species. Our docking results could confirm that the hydroxyl group of collagen could directly bind several amino acids on GPVI, and possibly stabilize the protein complex. However, we also found that the K^61^ residue is not involved in the hydroxyl groups or GPO-binding (Supplementary Figure S8), supporting our hypothesis that the observed amino acid substitution (K/**Q**, K/**E**, or K/**N**) may influence collagen-binding to GPVI in different species. However, this mechanism is independent of GPO-binding. 

Studies with the inhibitory monoclonal antibody JAQ-1, which induces irreversible internalization and shedding of the GPVI receptor [11,41], indicated the existence of two collagen-binding sites on GPVI, and the primary site overlaps with the CRP site [44]. Using a combinatorial library of linear pentadecamer peptides by phage display technology, Lecut et al. screened monoclonal antibodies (mABs) to identify the binding epitopes of collagen on GPVI. Interestingly, 9O12 mAB binds several phage peptide sequences (1:SCGLGVVCGAALVA, 2:GQELLACGLFSVCLS, 3:GQRSSVGGCGLHLVC, and 4:KNGVFLCGLGLVCPD) which correlated with a consensus motif of CGLxxVC. This conserved motif was not found in human GPVI. However, they proposed a weak homology region on human GPVI (^41^EKPVTLRCQGPPGVDLYRLE^60^), suggesting that this amino acid sequence may be a collagen- and/or CRP-binding epitope of GPVI [23]. Furthermore, they showed that 9O12 mAB only recognized a functional epitope, since the 9O12 mAB only recognizes GPVI under non-reducing condition in western blot experiments [23,45]. Using site-directed mutagenesis and a heterologous system to overexpress several mutant variants of GPVI, they could identify that mutation of G^50^, V^54^, and L^56^ to alanine in human GPVI could inhibit collagen- or CRP-binding, to different degrees, in certain experimental conditions [23]. Interestingly, the 9O12 mAB could bind all these mutant forms of GPVI, but the affinity was highly variable. Therefore, they concluded that (i) unknown residues, other than V54 and L56, seem to be part of the GPVI epitope for 9O12 mAB, and (ii) V54 and L56 are critically involved in GPVI interaction with collagen and CRP [23,46].

Interestingly, these indicated amino acids exist in our peptide (^48^CQ**G**PPG**V**D**L**YRLEKL^62^), used for the generation of the EA-20 antibody. We further analyzed the possible contribution of residues 48-55 (^48^CQGPPGVD^55^) to the GPVI-collagen binding. We found that these residues have no direct contact with collagen, (Supplementary Figure S9), while 56–62 residues (^56^LYRLEKL^62^) are directly involved in collagen binding. Nevertheless, intramolecular interactions of amino acid residues, such as hydrogen bond and hydrophobic interactions, could maintain the secondary structure and proper folding of this peptide sequence. Amino acid residues of ^48^CQGPPGVD^55^ show larger contact area, which probably supports the interaction between the EA-20 antibody and GPVI. 

Recently, the humanized monoclonal antibody glenzocimab (ACT017), which is a derivative of the 9O12 IgG antibody, was positively evaluated in clinical trials (Phase1b/2a studies), showing reduced intracerebral hemorrhage and mortality in patients with stroke [28,47]. In contrast to the previous study with the 9O12 antibody, crystallization studies mapped the glenzocimab-binding site in the D2 domain of GPVI, which is far away from the collagen-binding site [48]. Furthermore, glenzocimab inhibited GPVI function by preventing GPVI dimerization and inducing steric hindrance that prohibited binding to adjacent GPVI-binding sites on collagen, CRP, and fibrin [48]. 

Horii et al. showed a putative CRP-binding site using two different computational algorithms (PatchDock and FTDock) [12]. Both docking programs positioned the CRP-binding site of GPVI within the D1 domain involving several amino acid residues (^63^**SS**S**R**Y**Q**D**Q**AV**LF**I**P**AM**K**R**SL**^82^), located at close proximity to our peptide sequence that we used for the generation of the EA-20 antibody, although it is not overlapped. Nevertheless, they also reported that residues of V54 and L56 might form a secondary binding site for CRP and collagen. 

Another group used a three-dimensional model of the human D1/D2 domain of GPVI. This approach was based on the crystal structures of the killer Ig-like receptors, which showed a homologues structure to GPVI [21]. Using site-directed mutagenesis of GPVI, they identified the amino acid residue K^79^ (^63^SSSRYQDQAVLFIPAM**K**RSL^82^) which interacted with the inhibitory single-chain antibody fragment (scFv) of the 10B12 antibody [21]. This result suggests that the collagen-binding site of GPVI is located in close proximity to the amino acid of K^79^.

Recently, Feitsma et al. published the crystal structure of GPVI interacting with collagen or CRP. We recognized that the structure of GPVI in complex with CRP (PDB code: 5OU8) and the dimeric conformation of GPVI (PDB code: 2GI7), which we received from the protein structure databank (http://www.rcsb.org), were similar to that published in Feitsma et al., where the authors also identified the ligand-bound structure on the D1 domain of GPVI [30]. Using solid-state assays with mutant variants of GPVI, they could identify several amino acid residues essential for collagen- and CRP-binding. Two amino acid residues (R^58^ and E^60^) are located on the minimal collagen-binding epitope detected by the EA-20 antibody (^48^CQGPPGVDLY**R**L**E**KL^62^), and mutations of these residues could strongly reduce ligand-binding of GPVI. Interestingly, a similar epitope was also observed in the human leukocyte-associated Ig-like receptor (LAIR-1), and mutation of this site also reduced collagen binding [30]. Furthermore, at close proximity to the minimal epitope detected by the EA-20 antibody, Y^67^ and D^69^ mutations (^63^SSSR**Y**Q**D**QAVLFIPAMKRSL^82^) moderately changed the biochemical properties of GPVI. Based on the crystal structure of GPVI, the current model showed that the active form of GPVI does not change conformation after ligand binding, supporting the idea that the collagen-binding surface on GPVI is similar in resting and activated platelets [30]. They could also confirm earlier published results by Horii et al, showing distinct sites between dimerization (D2 domain) and collagen binding (D1 domain) [30]. 

It is still an open question whether receptor clustering or dimerization is a necessary step for GPVI activation. Our docking results suggest that dimers could effectively bind collagen fibers, including symmetric amino acid residues located on the D1 domain of each monomer. We also agree that dimerization seems to not change the conformation of the collagen-binding surface of GPVI; therefore, repetitive GPO and hydroxyl-groups of collagen can spontaneously interact with GPVI dimers. We speculate that dimers of GPVI form a cluster around the collagen fibers, which is an essential step in stabilizing the protein complex. Monomeric GPVI can form fewer bridges with hydroxyl-groups and GPO motifs, which may be not sufficient to keep collagen fibers on the platelet surface under shear condition, but may effectively bind collagen at static condition.

The crystal structure of human GPVI identified by Feitsma et al. opens a gate for an in silico docking study to identify novel drugs which may selectively inhibit interaction between GPVI and collagen. We could experimentally prove Feitsma et al.’s model, using our functional blocking EA-20 antibody, which specifically inhibits collagen and CRP interactions with GPVI. As a proof of principle, we evaluated its inhibitory effects on collagen-induced platelet adhesion, activation, and thrombus growth. 

Experimental studies also highlighted the relevance of targeting of GPVI-binding with collagen or collagen-like domain coding proteins in arterial thrombosis, ischemic stroke, and tumor metastasis [28,49,50,51,52]. Although recent studies highlighted GPVI as a safe-anti thrombotic target in patients with stroke, site-specific targeting approaches for GPVI are still missing. Future investigation is necessary to evaluate the antithrombotic potential of ^48^CQGPPGVDLYRLEKL^62^ minimal epitope blockade in vivo using a humanized GPVI mouse model. To design an anti-GPVI strategy in vivo, monoclonal humanized (hmAB) variants of EA-20 antibody should be generated to better understand the pathological impact of collagen-rich prothrombotic environment and validate the efficacy of the hmAB variant of EA-20 antibody at the preclinical level using a humanized GPVI mouse model.

In summary, we identified the minimal collagen and CRP-binding epitope of GPVI (^48^CQGPPGVDLYRLEKL^62^), and found that blocking this epitope does not influence fibrin binding of mouse and human platelets. Our study may help to design safe antithrombotic drugs which selectively inhibit GPVI interaction with collagen, thereby inhibiting thrombosis without affecting fibrin-controlled hemostasis. 

## Figures and Tables

**Table 1:** Binding mode of collagen with GPVI. * Symbol depicts residues which are in the peptide (CQGPPGVDLYRLEKL). The closest distances are presented.

Residue	Closest Residues	Distance, Å	Specific Interactions
GPVI:58:Arg *	collagen-1 : 4:Gly	2.7	1x hb to collagen-1 : 4:Gly
	collagen-1 : 3:Hyp	3.0	1x hb to collagen-1 : 3:Hyp
	collagen-1 : 6:Hyp	3.2	
	collagen-2 : 5:Pro	3.3	
	collagen-2 : 4:Gly	3.9	
GPVI:60:Glu *	collagen-1 : 6:Hyp	2.8	2x clash to collagen-1 : 6:Hyp
	collagen-2 : 5:Pro	3.4	
GPVI:62:Leu *	collagen-2 : 8:Pro	4.0	
**GPVI:67:Tyr**	collagen-1 : 6:Hyp	3.3	
	collagen-1 : 3:Hyp	3.7	
	collagen-1 : 5:Pro	3.7	
	collagen-1 : 4:Gly	4.0	
**GPVI:87:Arg**	collagen-2 : 6:Hyp	3.1	2x hb to collagen-2 : 6:Hyp
	collagen-2 : 7:Gly	3.2	
	collagen-2 : 8:Pro	3.2	
**GPVI:89:Ser**	collagen-2 : 5:Pro	3.7	
**GPVI:91:Gln**	collagen-1 : 3:Hyp	2.7	1x hb to collagen-1 : 3:Hyp
	collagen-2 : 2:Pro	3.9	
**GPVI:96:Trp**	collagen-2 : 3:Hyp	3.2	1x hb to collagen-2 : 3:Hyp
	collagen-2 : 5:Pro	3.8	
	collagen-2 : 4:Gly	3.8	
	collagen-1 : 3:Hyp	3.8	

## Data Availability

All data are included in the manuscript as main figures, tables, or Supplementary Materials.

## Supplementary Materials

The following supporting information can be downloaded at: https://www.mdpi.com/article/10.3390/biomedicines11020423/s1.
