# Supplementary material for: Minimal Collagen-Binding Epitope of Glycoprotein VI in Human and Mouse Platelets
Source: Biomedicines. 2023 Feb 1;11(2):423. doi: 10.3390/biomedicines11020423 (PMC9952969; doi:10.3390/biomedicines11020423)
Supplement: Supplementary file 1 [file biomedicines-11-00423-s001.zip › biomedicines-2141394-supplementary.pdf]

A

B

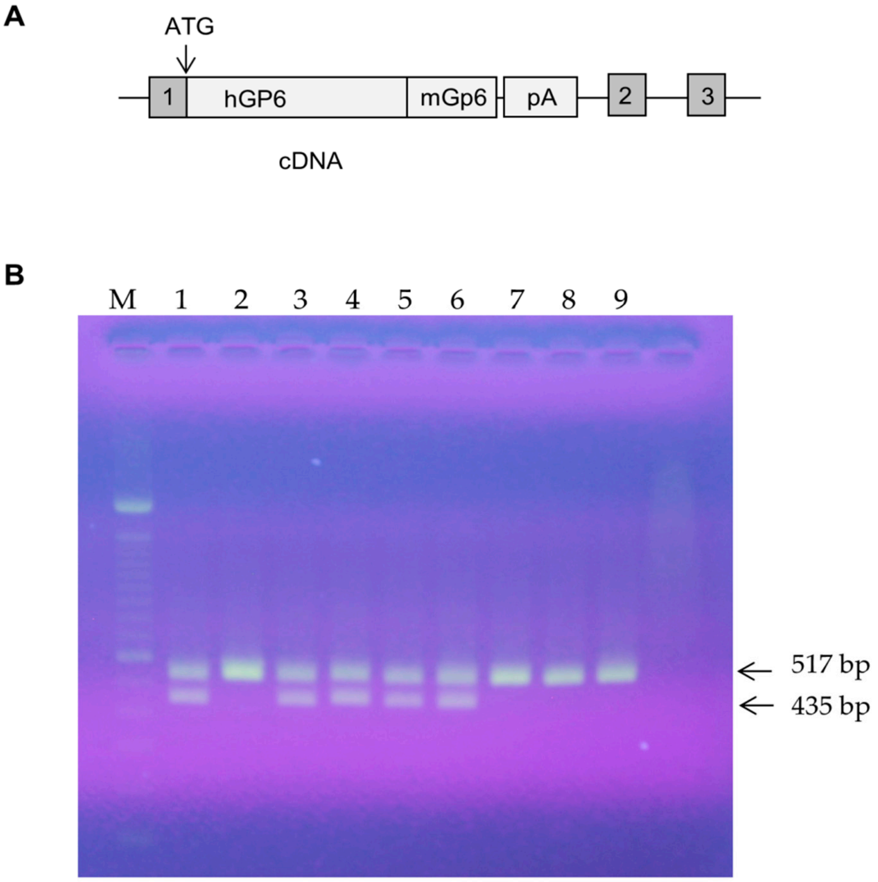

Supplementary Figure S1. (A) Modification of mouse GPVI gene. Insertion of chimeric cDNA of *hGP6* is indicated. Exons are in gray box, cDNA and polyA are in white box. Exon1 and cDNA were fused at ATG (B) Genotyping of *hGP6* and wild-type mice by PCR. Representative picture of agarose gel shows genotypes of homozygous and heterozygous mice (wild-type: 435 bp, *hGP6*: 517 bp).

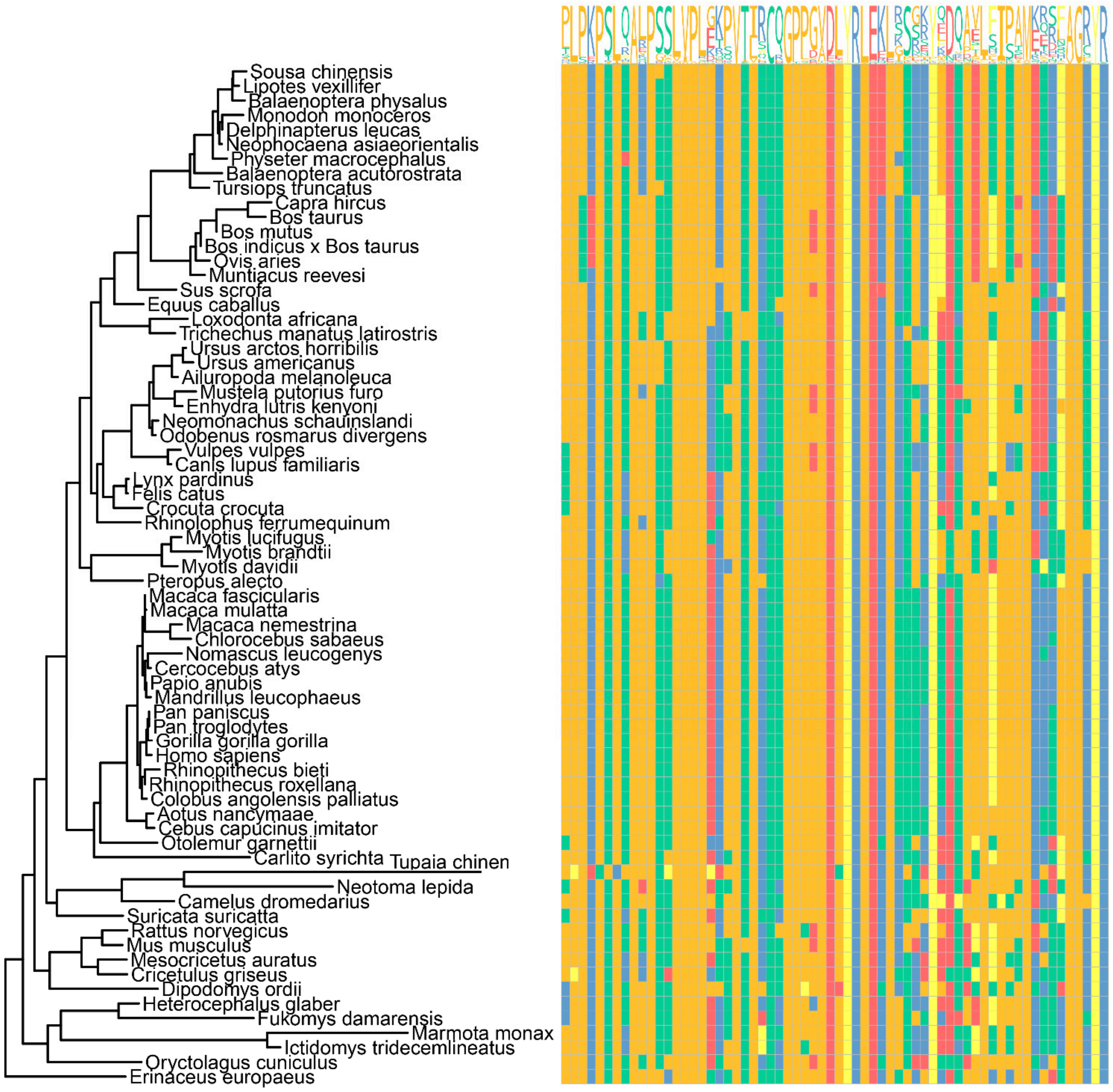

Supplementary Figure S2. Phylogenetic analysis for GPVI D1. The amino acid sequence of GPVI D1 were obtained from UniProt. The sequences were then aligned by ClustalW multiple alignments. The phylogenetic hierarchical clustering was constructed using the neighbor joining tree method with 1000 bootstrap replications for evaluating the reliability of the analysis. Colored columns represent the corresponding amino acids.

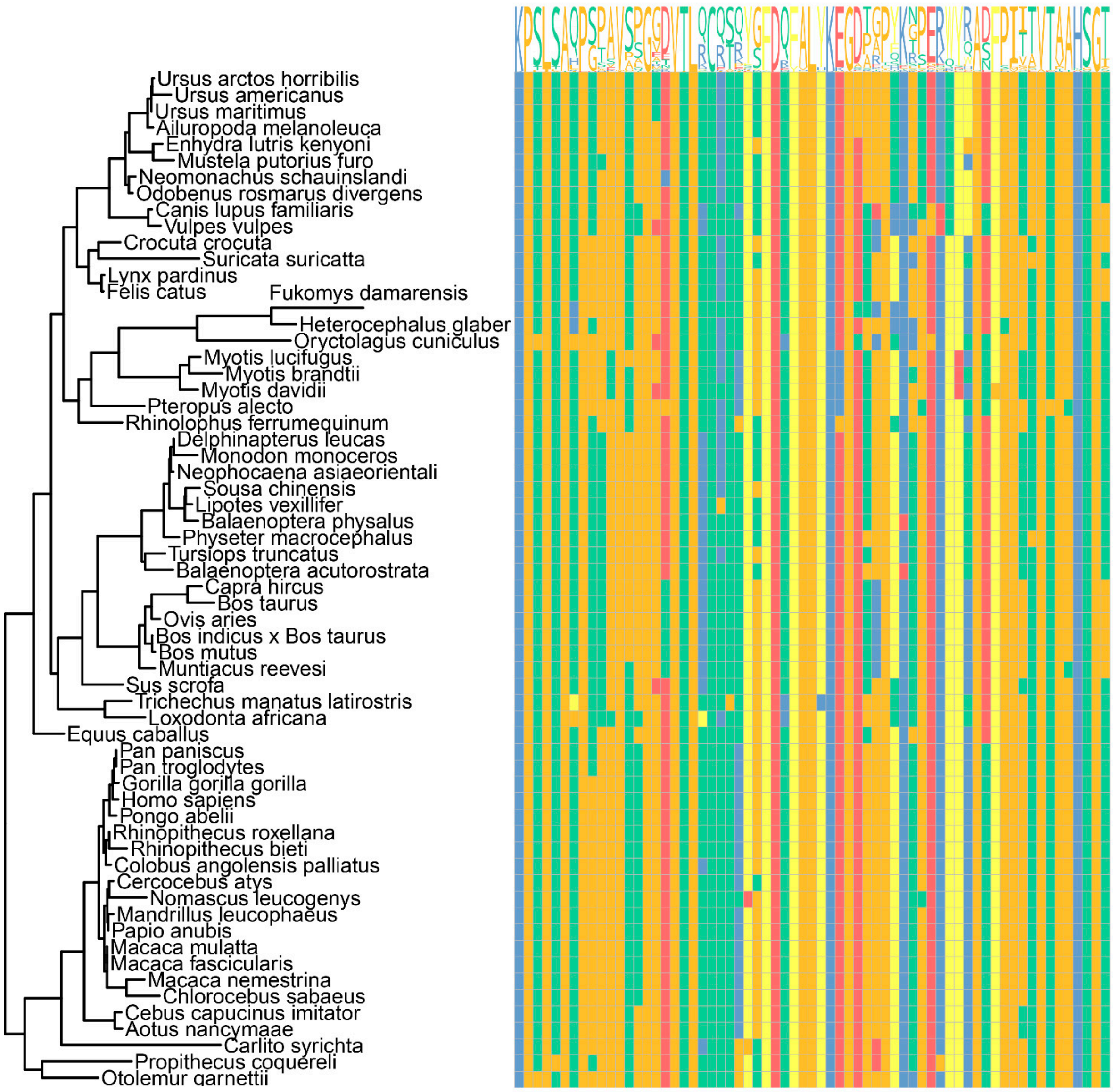

Supplementary Figure S3. Phylogenetic analysis for GPVI D2. The amino acid sequence of GPVI D2 were obtained from UniProt. The sequences were then aligned by ClustalW multiple alignments. The phylogenetic hierarchical clustering was constructed using the neighbor joining tree method with 1000 bootstrap replications for evaluating the reliability of the analysis. Colored columns represent the corresponding amino acids.

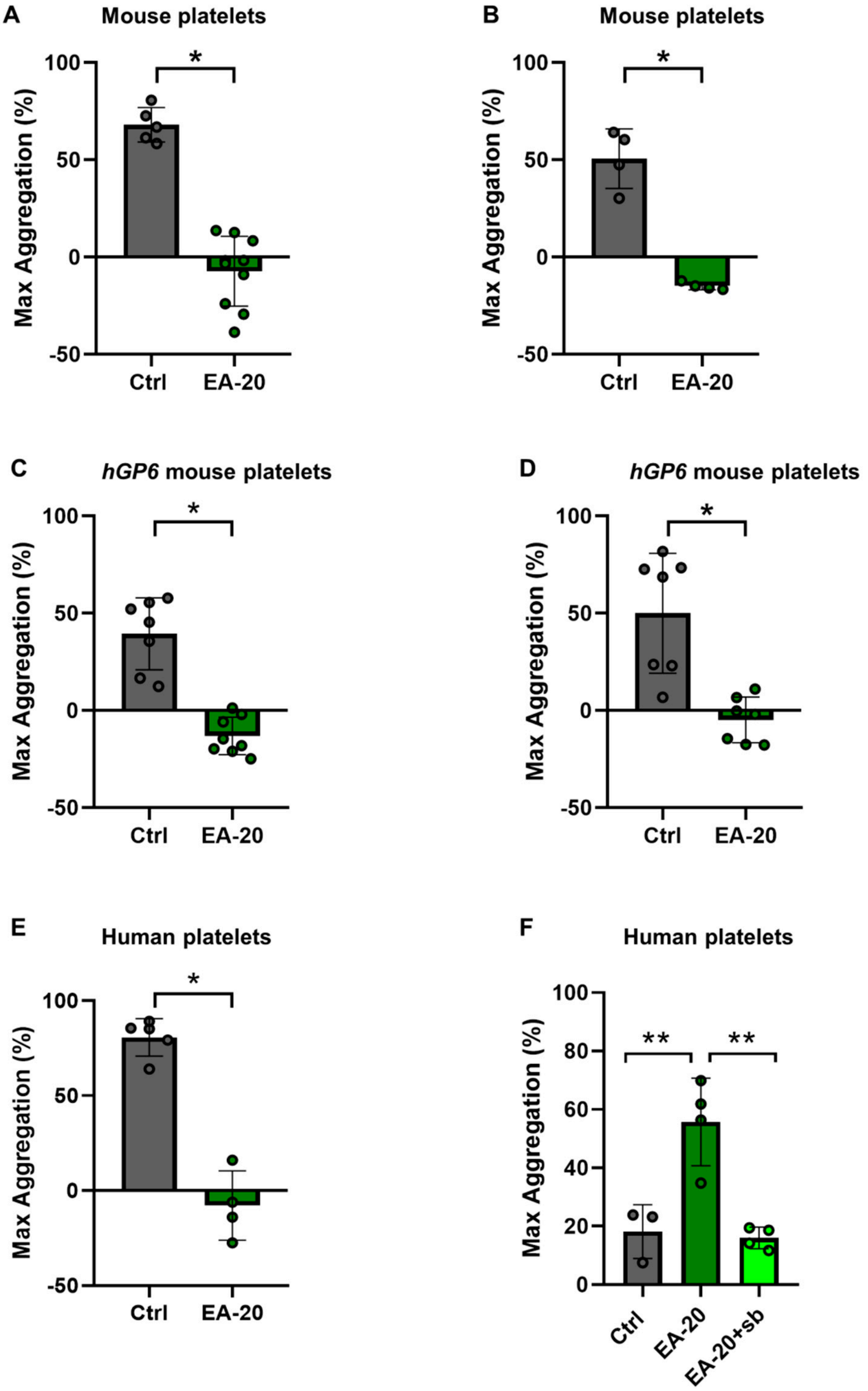

Supplementary Figure S4. EA-20 antibody impairs aggregation of mouse and human platelets in response to Col-I and CRP. Washed mouse and human platelets were stimulated with Col-I (**A, C, E** Col-I 2.5 µg/mL) and CRP (**B, D** CRP 0.2 µg/mL and **F** CRP 0.4 µg/mL) in the presence of control IgG (Ctrl) or EA-20 antibody. Experiments were performed using (**A, B**) *WT* mouse, (**C, D**) humanized GPVI (*hGP6*) mouse and (**E, F**) human platelets. Platelet aggregation was measured using light transmission aggregometry and recorded for 10 min. (**A-F**) Mean ± standard deviation (SD), Ctrl (IgG control antibody) and EA-20. Each point represents one individual mouse or healthy human donor. Mann-Whitney U test, *$P < 0.05$ and 1-way ANOVA and Tukey's post hoc test, **$P < 0.01$.

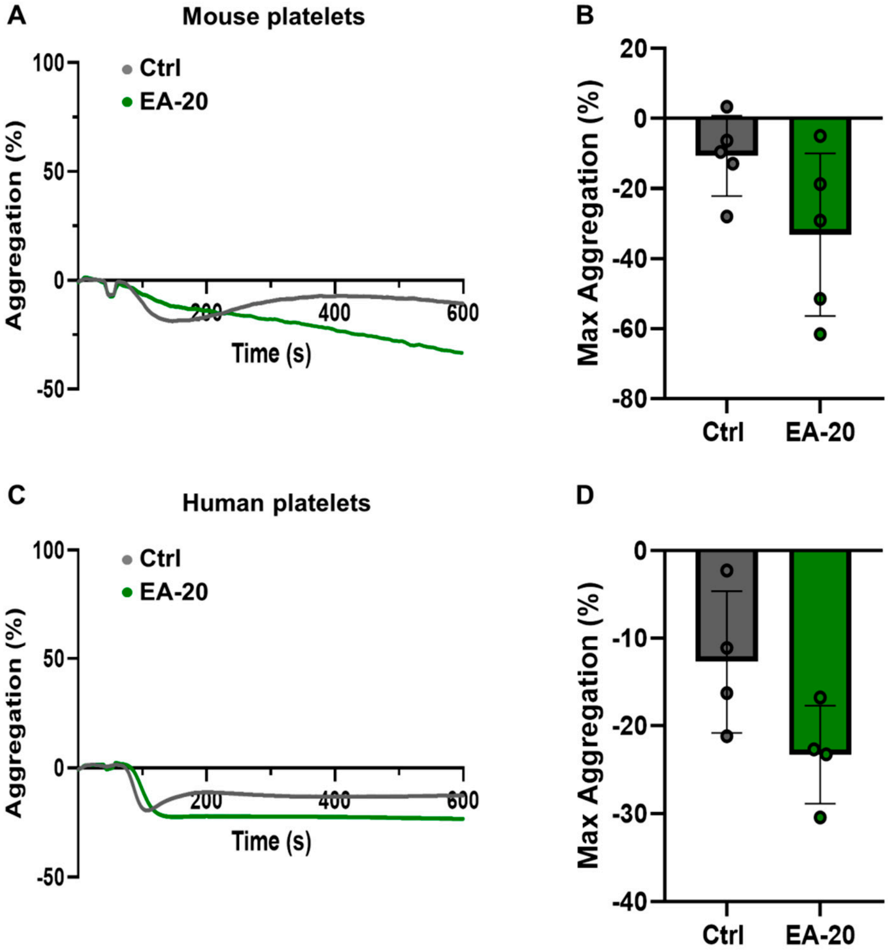

Supplementary Figure S5. Effect of EA-20 antibody on aggregation of mouse and human platelets in response to low dose of Col-I. Washed mouse and human platelets were stimulated with Col-I (**A, B** Col-I 0.2µg/mL and **C, D** Col-I 0.5µg/mL) in the presence of control IgG (Ctrl) or EA-20 antibody. Platelet aggregation was measured using light transmission aggregometry and recorded for 10 min. Representative aggregation curves are shown. Mean ± standard deviation (SD), Ctrl (IgG control antibody) and EA-20. Each point represents one individual (**B**) mouse or (**D**) healthy human donor.

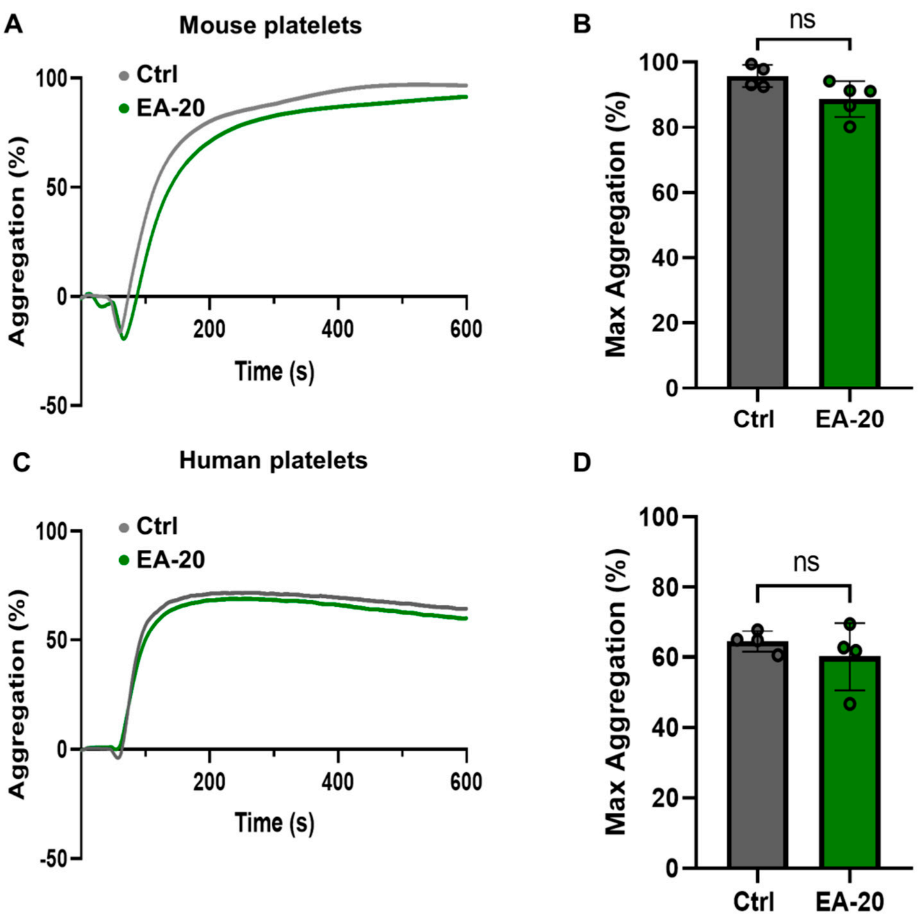

Supplementary Figure S6. Effect of EA-20 antibody on aggregation of mouse and human platelets in response to thrombin and TRAP-6. Washed mouse and human platelets were stimulated with thrombin (**A, B** Thrombin 0.01U) and TRAP-6 (**C, D** TRAP-6 10μM) in the presence of control IgG (Ctrl) or EA-20 antibody. Platelet aggregation was measured using light transmission aggregometry and recorded for 10 min. Mean ± standard deviation (SD), Ctrl (IgG control antibody) and EA-20. Each point represents one individual (**B**) mouse or (**D**) healthy human donor. Mann-Whitney U test, ns: not significant.

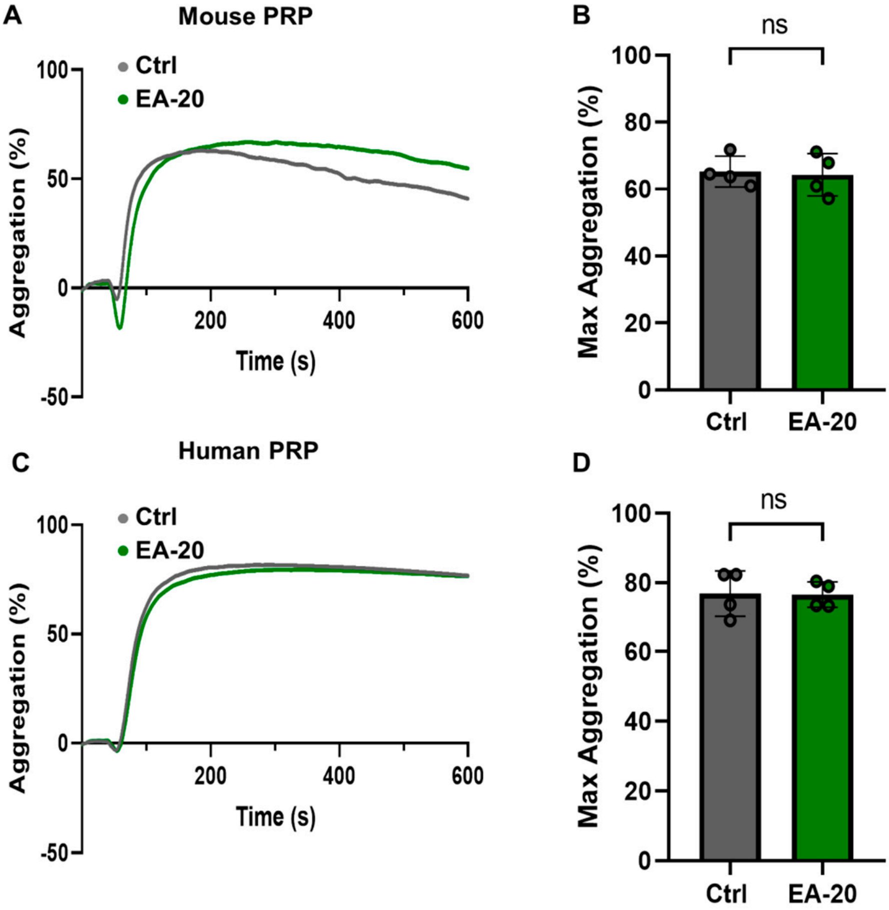

Supplementary Figure S7. Effect of EA-20 antibody on aggregation of mouse and human platelets in response to ADP. Washed mouse and human PRP were stimulated with ADP (10µM) in the presence of control IgG (Ctrl) or EA-20 antibody. Platelet aggregation was measured using light transmission aggregometry and recorded for 10 min. Mean ± standard deviation (SD), Ctrl (IgG control antibody) and EA-20. Each point represents one individual (**A, B**) mouse or (**C, D**) healthy human donor. Mann-Whitney U test, ns: non-significant.

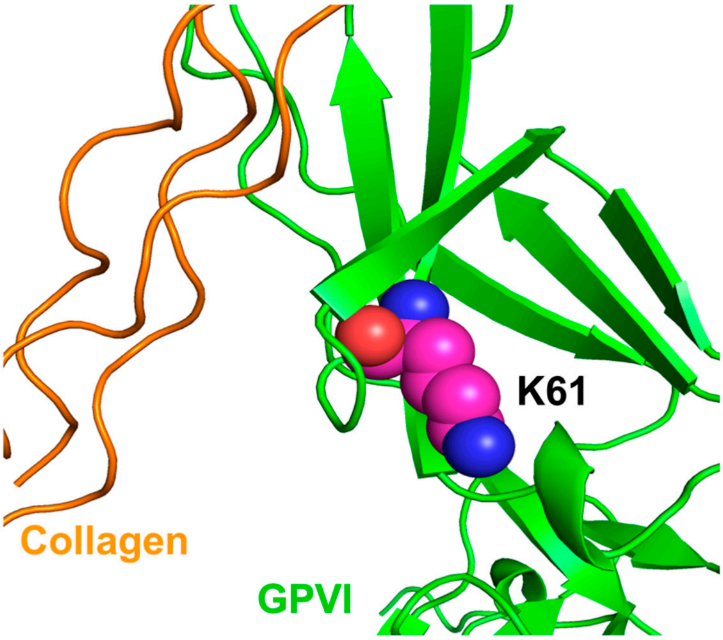

Supplementary Figure S8. Interaction between collagen and K61 residue of GPVI. The complex structure (PDB code: 5OU8) of GPVI and collagen-peptide are retrieved from the protein structure databank. Collagen peptides are shown in orange, GPVI is shown in green and K61 depicted as sphere.

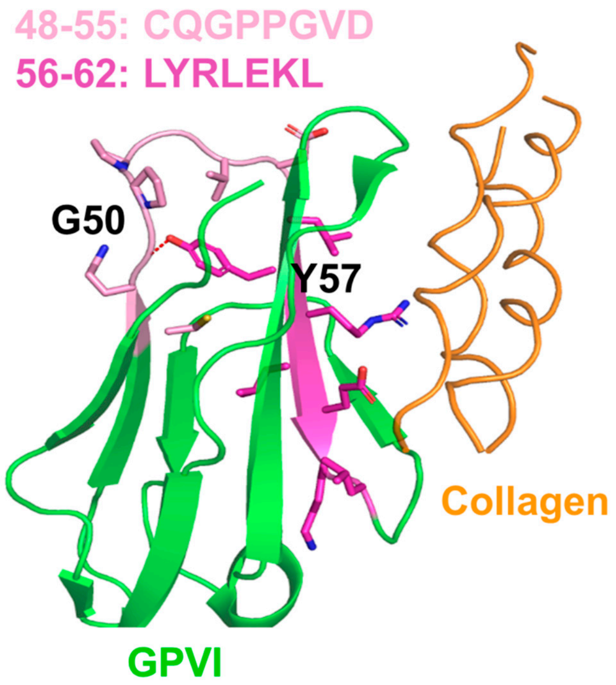

Supplementary Figure S9. High resolution of the minimal collagen-binding epitope of GPVI. GPVI was shown in green, peptide residues 48-55 in GPVI, peptide residues 56-62 in GPVI, and collagen are shown in pink, magenta and orange, respectively. The interaction residues are depicted as sticks. Red dash lines indicate hydrogen bonds between residues.
